# Complications of Untreated Advanced HIV/AIDS With Kaposi Sarcoma in a Young African American Male: A Case Report

**DOI:** 10.7759/cureus.69449

**Published:** 2024-09-15

**Authors:** Brittney Koruthu, Ali Z Ansari, Sahar Hafeez, Shivum Desai, Dhruv U Patel, Srihita Patibandla, Nilay Bhatt

**Affiliations:** 1 Department of Pathology, William Carey University College of Osteopathic Medicine, Hattiesburg, USA; 2 Department of Emergency Medicine, University of Mississippi Medical Center, Jackson, USA; 3 Department of Internal Medicine, Trinity Health Grand Rapids, Grand Rapids, USA; 4 Department of Internal Medicine, Merit Health Wesley, Hattiesburg, USA

**Keywords:** antiretroviral therapies, cutaneous lesion, hiv-positive, human herpesvirus 8 (hhv-8), immunosuppression complications, kaposi sarcoma management, neoplastic disease, pleural effusions, spindle-cell tumor, young adult male

## Abstract

Kaposi sarcoma (KS) represents a neoplastic proliferation primarily affecting endothelial cells, characterized by the development of cutaneous lesions. However, its pathogenesis can extend beyond the skin, involving internal organs, lymph nodes, and mucous membranes. KS is associated with human herpesvirus 8 (HHV-8) and is often prevalent in immunocompromised patients, especially those with human immunodeficiency virus (HIV) and/or acquired immunodeficiency syndrome (AIDS). We present a case of a 29-year-old African American male who came into the emergency department (ED) with overall discomfort and a boil on his buttock, which was later found to be symptoms of KS in the context of progressed HIV infection. This case report highlights the complications that arise when HIV/AIDS is left untreated and subsequently leads to the development of KS. Early recognition and appropriate interventions are crucial in optimizing outcomes and guiding future care decisions.

## Introduction

Kaposi sarcoma (KS) is a type of soft tissue neoplasm that is most commonly associated with human herpesvirus 8 (HHV-8) [1]. Hence, it is also known as KS-associated herpesvirus. It commonly occurs in patients with immunosuppression, such as those with acquired immunodeficiency syndrome (AIDS) or those undergoing immunosuppressive therapy due to an organ transplant. Despite its global presence, the incidence of KS varies significantly, being notably higher in sub-Saharan African populations and among individuals with human immunodeficiency virus (HIV) and AIDS, reflecting the critical interplay between epidemiological factors and immunosuppression [2]. This cancer often manifests as cutaneous lesions but may extend to involve diverse organ systems. For example, in the lungs, it can present with pulmonary consolidation and infiltrates.

Clinically, KS often presents with distinctive red or purple plaques or nodules on cutaneous or mucosal surfaces. The lesions can be associated with marked edema and may vary in size and distribution. Beyond the skin, KS can involve various organ systems, including the lungs, where it may present with pulmonary consolidation and infiltrates. Involvement of internal organs often signifies more advanced disease [3]. Differential diagnosis for KS includes other vascular lesions such as hemangiomas and angiosarcomas and other conditions that may present with cutaneous nodules or plaques, including metastatic disease and certain dermatologic conditions.

Pathologically, KS progresses through three distinct stages: patch, plaque, and nodule [4]. Grossly, the patch stage presents flat, slightly raised lesions with a reddish hue. Histologically, it is characterized by a spindle cell proliferation of irregular, complex vascular channels dissecting through the dermis, with the promontory defined as proliferating vessels surrounding larger ecstatic pre-existing vessels and skin adnexa. In the plaque stage, lesions become thicker and more elevated, with a deeper reddish-purple color. Histologically, features are increased in prominence from the patch stage, with extension into the subcutis and a greater presence of hyaline globules both intracellularly and extracellularly. In the nodule stage, the lesions present as firm raised nodules with a violaceous hue. Histologically, pleomorphism increases and mitotic figures become more prominent, indicating more aggressive disease progression [4]. The immunocompromised state induced by HIV/AIDS not only predisposes individuals to KS but also accelerates its progression, emphasizing the disease's aggressive nature in this population.

Furthermore, the advent of antiretroviral therapy (ART) has significantly altered the landscape of KS, offering not only a reduction in incidence but also a potential avenue for management and prognosis improvement [5]. Treatment for KS depends on the type and extent of the disease. It may involve ART for HIV/AIDS patients, chemotherapy, immunotherapy, or other targeted therapies [6]. Early detection and appropriate medical management can improve outcomes for individuals with KS. In this case report, we highlight the complications that arise from untreated HIV/AIDS concurrent with KS in a young African American male.

## Case presentation

A 29-year-old African American male presented to the emergency department (ED) with a myriad of symptoms, including generalized body aches, joint pain, and shortness of breath that had been ongoing for months, along with persistent painful buttock swelling over the past week. The patient disclosed a known history of HIV, diagnosed two years ago, but had never received ART. The delay in ART initiation was due to personal and socioeconomic barriers, which the patient acknowledged during his history-taking. He had not followed up with a healthcare provider since his diagnosis, contributing to his lack of ART initiation. The patient was also unaware of his current CD4 count, which is a key indicator of immune system health in individuals with HIV. Additionally, the patient reported the development of oral thrush during the same period, a common fungal infection in individuals with untreated or advanced HIV due to a weakened immune system. Contributing factors included a smoking history equivalent to 4.5 pack-years, occasional social alcohol consumption, and past marijuana use several years ago. The patient denied symptoms such as fever, chills, nausea, vomiting, chest pain, abdominal pain, diarrhea, constipation, and dysuria, with no known sick contacts. He had tested negative for COVID-19 two weeks prior to the presentation.

**Figure 1:**
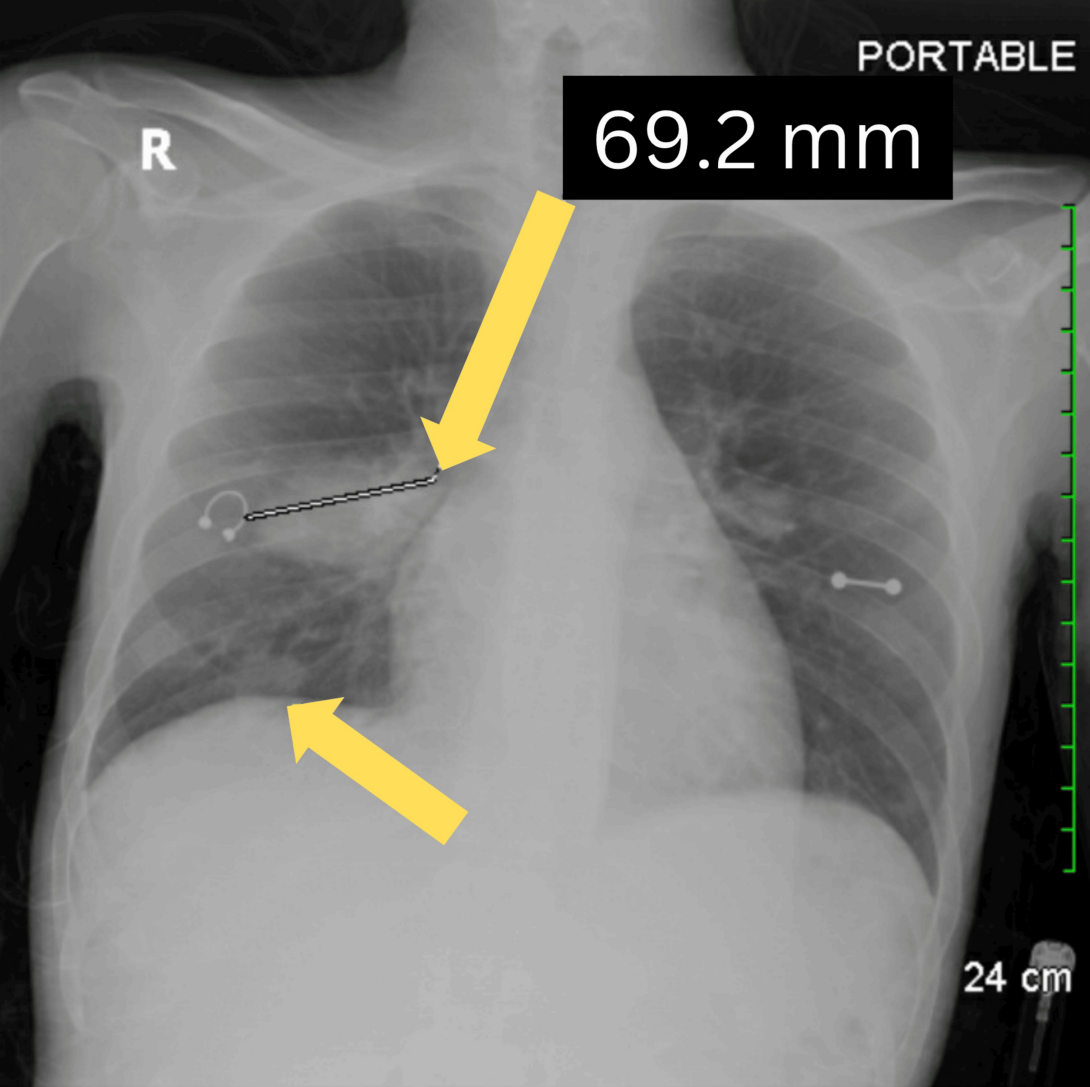
Chest x-ray revealing areas of density in the right mid-lung field and right lung base (yellow arrows).

During physical examination, slight compression of the swelling led to a serous discharge from the buttock swelling, and the patient complained of a sharp, non-radiating pain with an intensity of 10/10 on the pain scale. The patient also reported localized joint pain primarily involving the left hip and left wrist. His mouth was examined, and after scraping the tongue, no material came off, lowering the suspicion of an active fungal infection. A chest X-ray was performed due to the patient’s shortness of breath. The imaging revealed areas of increased density in the right mid-lung field and right lung base, which were identified as infiltrates (Figure 1). In the context of untreated HIV, these infiltrates may be indicative of pulmonary complications such as opportunistic infections or inflammatory processes. Such findings are common in individuals with advanced HIV due to their compromised immune system, which increases susceptibility to conditions like pneumonia or tuberculosis.

A focused ultrasound examination identified a subcutaneous hypoechoic area measuring 2.6 x 0.4 x 1.5 cm with peripheral hyperemia was noted in the right buttock. The finding consistent with a localized fluid collection was suggestive of a subcutaneous abscess. Evaluation of the left buttock revealed no evidence of abscess.

**Figure 2:**
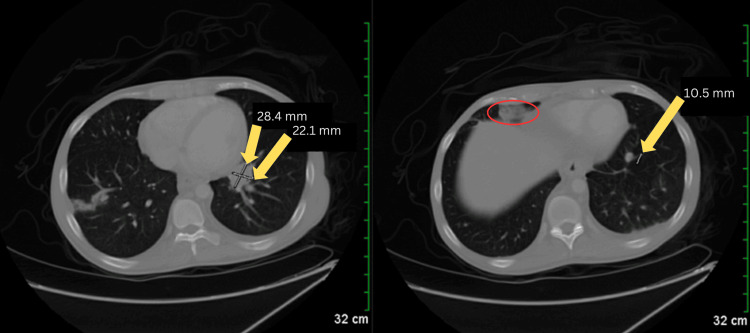
CT scan of the chest showing lung nodules and prominent lymphatic tissue (left, yellow arrows), along with a cyst in the pericardial region (right, red oval). CT: Computed tomography

**Figure 3:**
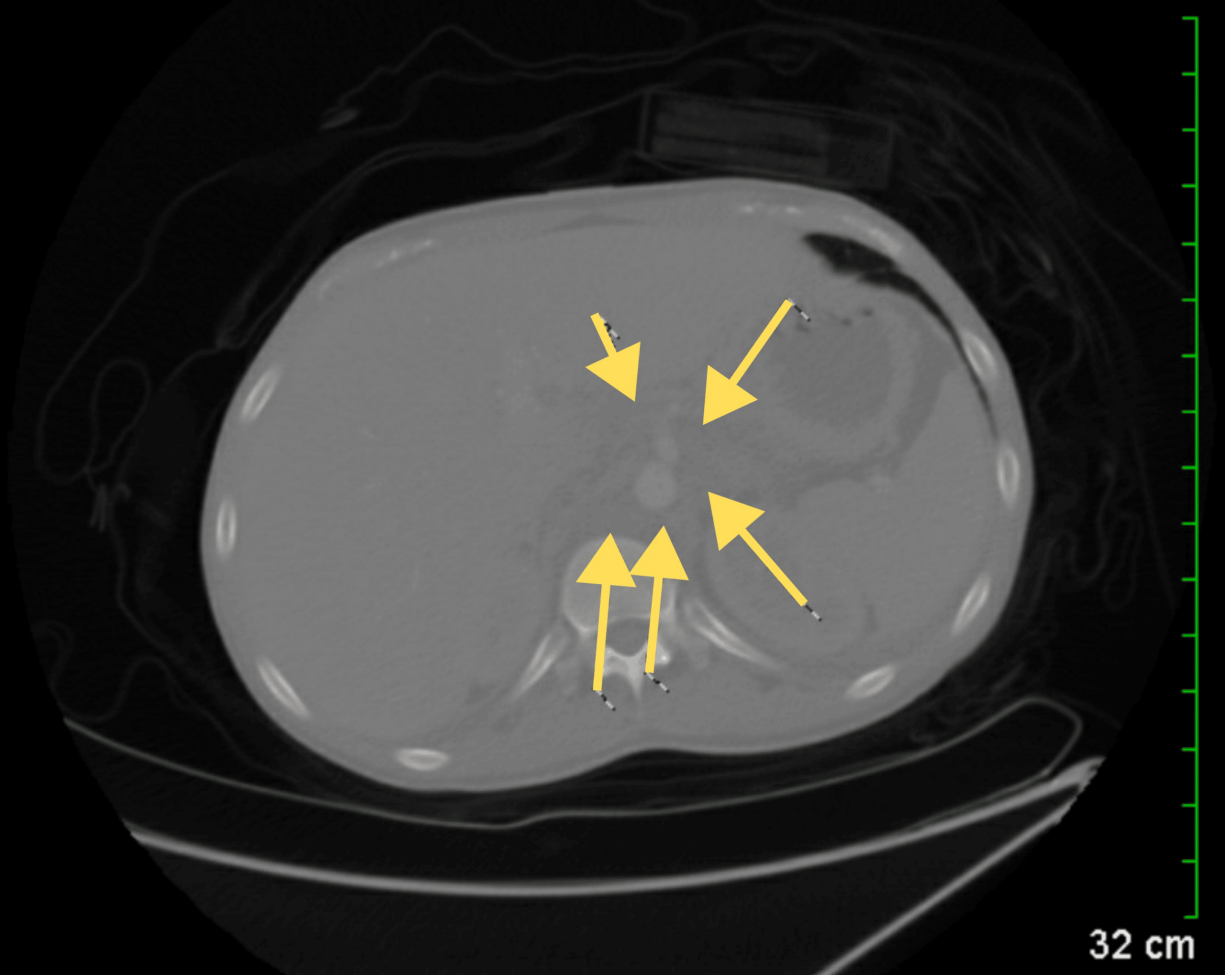
CT scan of the abdomen showing extensive confluent densities surrounding the aorta and centrally within the mesenteric fat (yellow arrows). CT: Computed tomography

**Figure 4:**
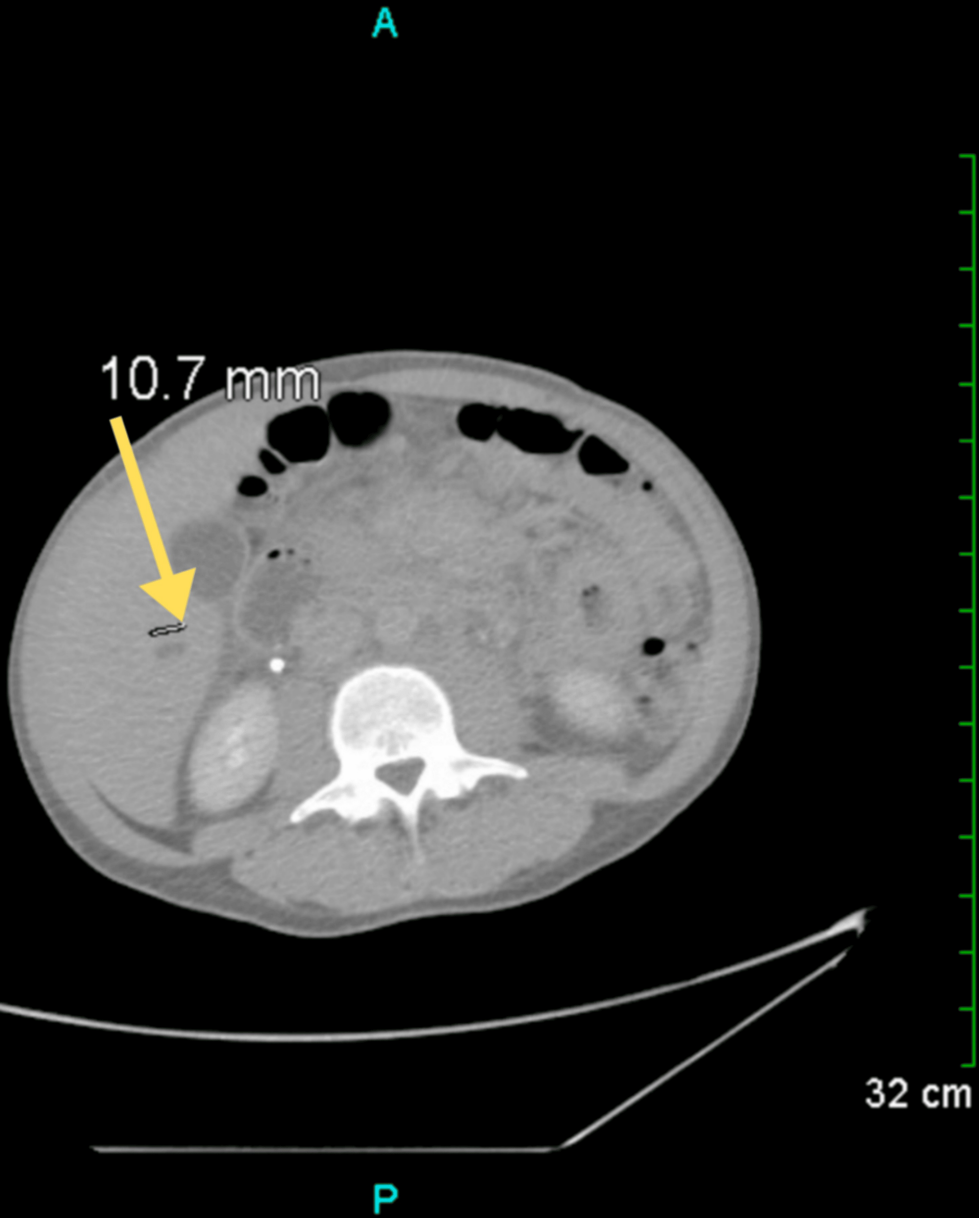
CT scan of the abdomen and pelvis revealing a small cyst in the liver (yellow arrow). CT: Computed tomography

Following the ultrasound examination, a computed tomography (CT) scan was performed, revealing new findings of lung nodules and prominent lymph tissue (Figure 2). CT scan of the chest and abdomen revealed extensive areas of confluent density in the periaortic region and centrally in the mesenteric fat planes of the abdomen, accompanied by scattered nodular and focal ill-defined densities in the lower lung fields (Figure 3). Additionally, adenopathy was noted in the left infra-hilar region. Given the patient's age and gender, lymphoma or metastatic testicular cancer was considered. On a separate CT of the abdomen and pelvis, minimal bilateral pleural effusions were detected, along with a small amount of free fluid in the right side of the abdomen in the lower pelvis. A small cyst was also observed in the liver (Figure 4).

Laboratory results revealed anemia, with a hemoglobin level of 5.7 g/dl, necessitating a blood transfusion. Additionally, a low reticulocyte count and elevated ferritin levels were noted. The patient underwent surgery the following day for incision and drainage and reported improvement postoperatively. Blood cultures conducted post-surgery revealed the presence of gram-positive cocci in chains.

**Figure 5:**
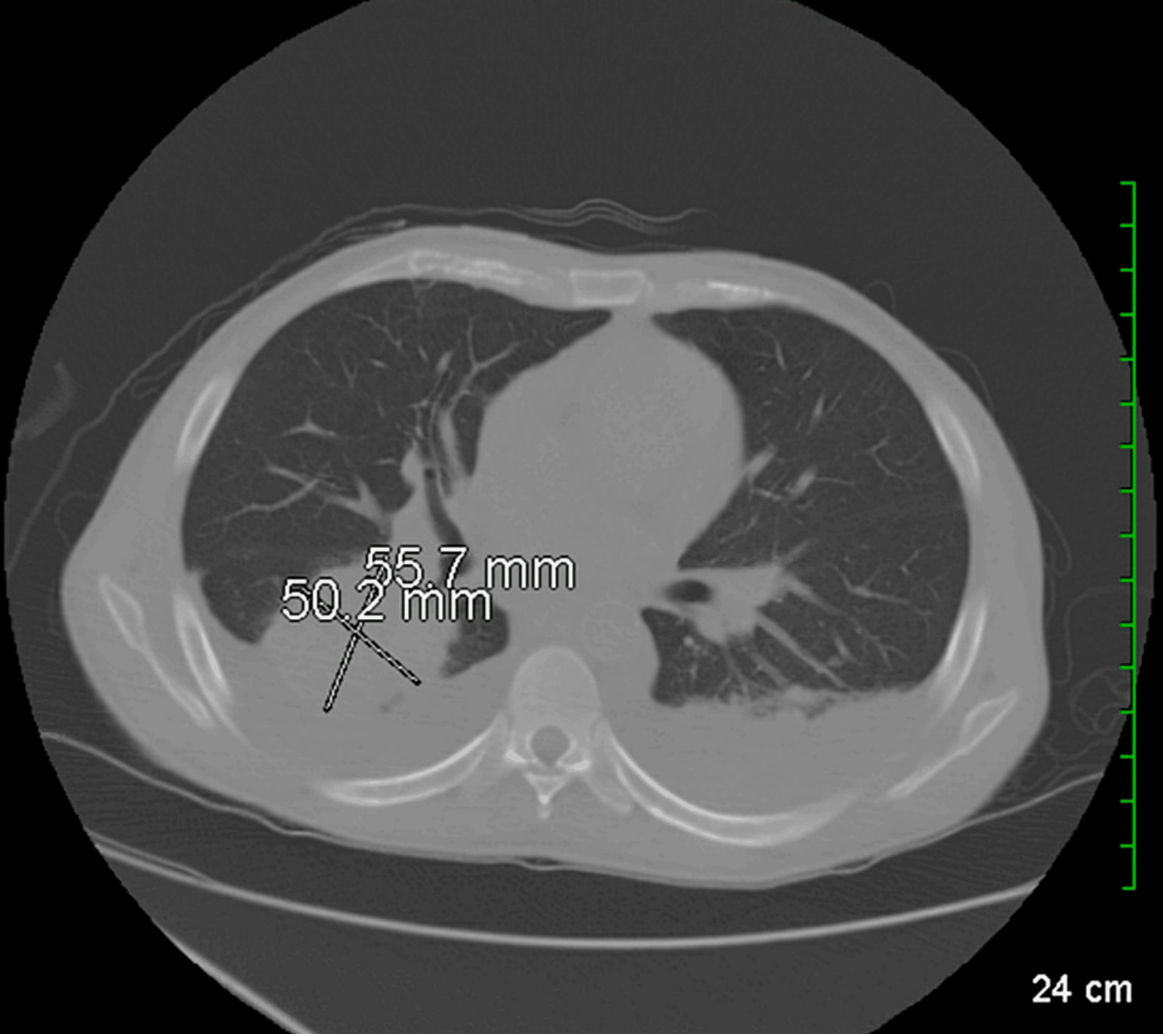
CT scan of the chest revealing multifocal infiltrates and mass-like consolidations. The image shows areas of increased density indicative of infiltrative processes, consistent with the presence of extensive pulmonary pathology CT: Computed tomography

The day after, a chest CT scan was conducted, with the patient reporting further improvement. The scan revealed multifocal infiltrates/mass-like consolidations, most notably in the right lower lobe, where an area of consolidation measured 5.6 x 5.0 cm (Figure 5). Additionally, smaller nodular consolidations, approximately a centimeter in size, were observed in the upper lobes. Bilateral effusions were also noted, which had significantly increased since the initial CT abdominal scan performed upon admission.

Three days later, a CT-guided biopsy of the chest was conducted. Pathological analysis of the mass revealed findings consistent with organizing pneumonitis, accompanied by recent hemorrhage and the presence of pigment-laden histiocytes. Immunohistochemical staining revealed 3+ positive expression of thyroid transition factor (TTF), Cam 5.2, and pankeratin in the cytoplasm and nucleus, with highlighting in the background breast parenchyma showing compression artifact. CD68 staining demonstrated rare clusters of histiocytes, while the iron stain was positive within the pigment cells, indicative of a remote hemorrhage. CD34 staining was diffusely positive, with a +3 intensity, favoring KS as the underlying etiology. Although further investigations, including specific immunohistochemical markers such as latency-associated nuclear antigen (LANA-1), platelet endothelial cell adhesion molecule (PECAM-1), D2-40, vascular endothelial growth factor receptor-3 (VEGFR-3), and B-cell lymphoma-2 (BCL-2), were considered, they were not performed due to resource limitations.

**Figure 6:**
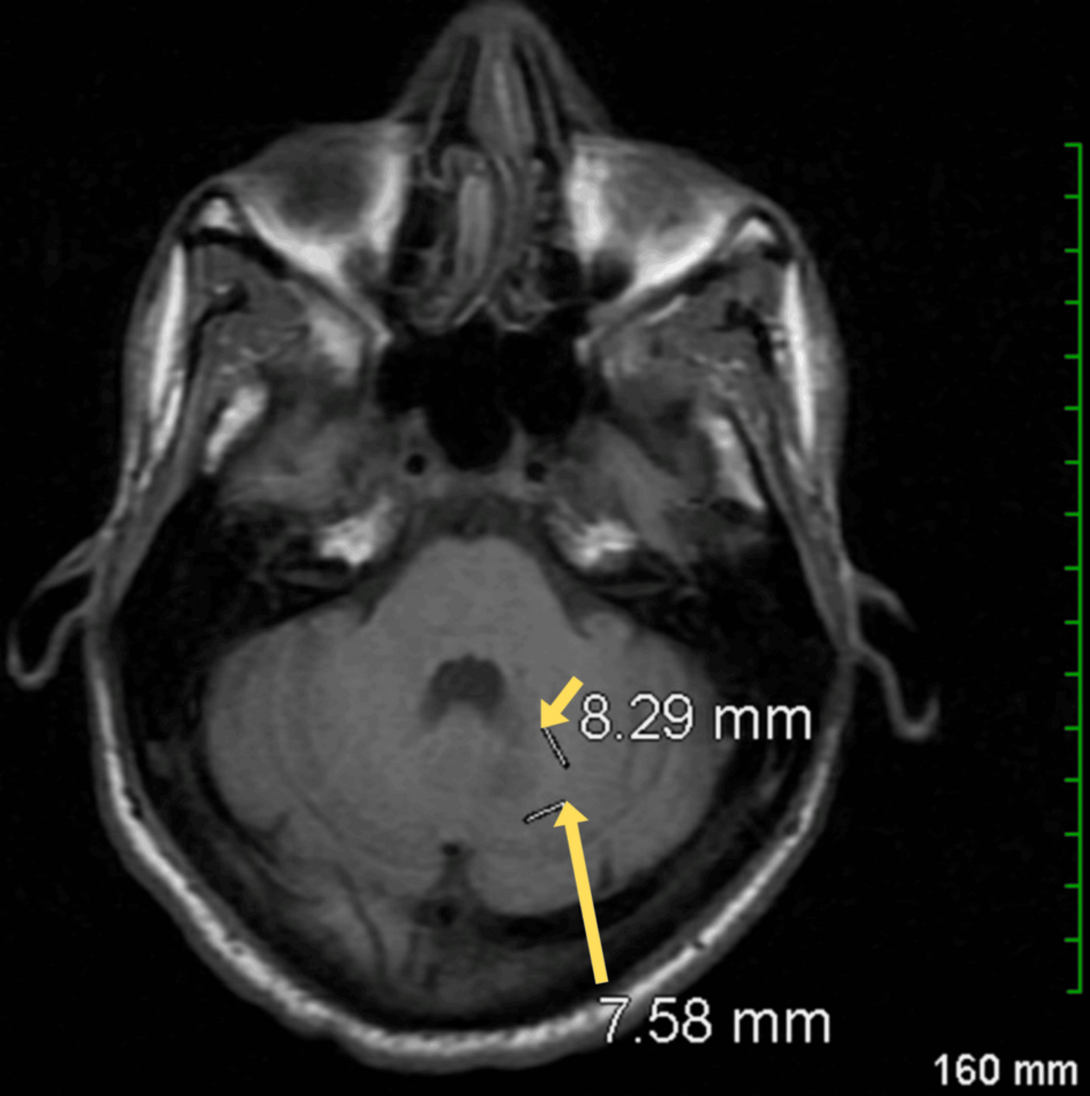
Brain MRI showing a focal area of restricted diffusion with enhancement and associated edema in the mid-medial left cerebellar hemisphere (yellow arrows). MRI: Magnetic resonance imaging

Further investigations revealed the presence of Epstein-Barr virus (EBV) DNA in the spinal fluid, which raised the possibility of an active EBV-related central nervous system (CNS) infection, such as primary CNS lymphoma or encephalitis. Brain magnetic resonance imaging (MRI) identified a focal area of restricted diffusion with enhancement in the mid-medial left cerebellar hemisphere, measuring 8 x 8 mm. This finding was accompanied by slight associated edema, though there was no effacement of the adjacent fourth ventricle (Figure 6). The restricted diffusion and enhancement suggest an inflammatory or neoplastic process. Given these findings, further evaluation for potential EBV-related CNS pathology was deemed necessary, and discussions about hospice care and future plans were initiated during follow-up for AIDS.

## Discussion

The patient's journey with KS likely followed a progression reflective of the disease's multifaceted nature. Initially, the patient experienced nonspecific symptoms such as generalized body aches and joint pain, which might have corresponded to the early stages of KS characterized by the development of flat or slightly raised lesions known as patches. As the disease advanced, the patient presented with a painful buttock swelling, suggestive of the transition to the plaque stage of KS, where lesions become thicker and more elevated [7]. The subsequent imaging findings of lung nodules and prominent lymph tissue, along with the pathological and IHC staining findings, indicated a more advanced stage of the disease, likely representing disseminated or systemic KS involving internal organs [8]. This progression highlights the aggressive nature of KS in the context of untreated HIV/AIDS, where the compromised immune system allows for widespread dissemination of the tumor [9].

The delay in initiating treatment for HIV/AIDS likely contributed to the progression of KS and the development of other complications, such as anemia requiring blood transfusion, pulmonary infiltrates, and neurological involvement, as evidenced by the presence of EBV DNA in the spinal fluid and cerebral lesions on MRI. ART plays a crucial role in suppressing HIV replication, restoring immune function, and reducing the risk of HIV-related complications, including KS [10]. However, the effectiveness of ART may be compromised when initiation is delayed, leading to disease progression and an increased risk of opportunistic infections and malignancies [11]. Moreover, the patient's lifestyle factors, including smoking and past marijuana use, may have further compromised his immune system and contributed to the severity of his illness.

Of particular concern was the delayed diagnosis of KS, as evidenced by the presence of subcutaneous hypoechoic areas and fluid collections consistent with abscess formation in the buttocks, as well as the subsequent discovery of lung nodules and prominent lymph tissue on imaging studies [12]. The presence of multifocal infiltrates and mass-like consolidations in the lungs, in conjunction with the other clinical and pathological findings, supports the diagnosis of disseminated KS [13]. The pathological analysis of the chest mass revealed findings consistent with organizing pneumonitis, accompanied by pigment-laden histiocytes and diffuse CD34 staining, further supporting the diagnosis of KS [14]. The initiation of ART at this stage could have had a significant impact on curbing the advancement of the disease by improving immune function and reducing the viral load, which are critical in managing HIV/AIDS and associated complications. This highlights the importance of a comprehensive diagnostic approach in patients with HIV/AIDS presenting with nonspecific symptoms, as timely recognition of KS and initiation of appropriate therapy can significantly impact patient outcomes.

## Conclusions

This case highlights the severe complications that can arise from untreated HIV/AIDS, particularly in the context of KS. The patient's presentation with advanced KS, characterized by cutaneous lesions, lung involvement, and systemic manifestations, emphasizes the aggressive nature of the disease in immunocompromised individuals. The delay in the diagnosis and management of both HIV/AIDS and KS as seen in this case illustrates the critical need for early detection and intervention to prevent disease progression and improve outcomes. Furthermore, lifestyle factors, such as smoking and past substance use, may have exacerbated the patient's immunosuppression, demonstrating the importance of comprehensive patient education and support in managing chronic conditions like HIV/AIDS. Clinicians should maintain a high index of suspicion for KS in HIV-positive patients presenting with nonspecific symptoms, especially when the patient is not on ART. A multidisciplinary approach, involving prompt diagnosis and appropriate management, is essential to optimize patient outcomes and enhance the quality of life for individuals living with HIV/AIDS.
